# HDAC6 Deficiency Has Moderate Effects on Behaviors and Parkinson’s Disease Pathology in Mice

**DOI:** 10.3390/ijms24129975

**Published:** 2023-06-09

**Authors:** Jiayin Zhao, Yongtao He, Yufei Duan, Yuanyuan Ma, Hongtian Dong, Xiaoshuang Zhang, Rong Fang, Yunhe Zhang, Mei Yu, Fang Huang

**Affiliations:** Department of Translational Neuroscience, Jing’an District Centre Hospital of Shanghai, State Key Laboratory of Medical Neurobiology and MOE Frontiers Center for Brain Science, Institutes of Brain Science, Fudan University, 138 Yixueyuan Road, Shanghai 200032, China; 22111520073@m.fudan.edu.cn (J.Z.);

**Keywords:** PD, behaviors, neuroinflammation, HDAC6

## Abstract

Histone deacetylase 6 (HDAC6) is involved in the regulation of protein aggregation and neuroinflammation, but its role in Parkinson’s disease (PD) remains controversial. In this study, *Hdac6*^−/−^ mice were generated by CRISPR-Cas9 technology for exploring the effect of HDAC6 on the pathological progression of PD. We found that male *Hdac6*^−/−^ mice exhibit hyperactivity and certain anxiety. In the acute 1-methyl-4-phenyl-1,2,3,6-tetrahydropyridine (MPTP)-induced PD mice, though motor injury was slightly alleviated by HDAC6 deficiency, dopamine (DA) depletion in the striatum, the decrease in the number of DA neurons in the substantia nigra (SN) and the reduction in DA neuronal terminals were not affected. In addition, activation of glial cells and the expression of α-synuclein, as well as the levels of apoptosis-related proteins in the nigrostriatal pathway, were not changed in MPTP-injected wild-type and *Hdac6*^−/−^ mice. Therefore, HDAC6 deficiency leads to moderate alterations of behaviors and Parkinson’s disease pathology in mice.

## 1. Introduction

Parkinson’s disease (PD), the second most prevalent neurodegenerative disorder, is characterized by a loss of dopaminergic neurons in the substantia nigra pars compacta (SNpc) accompanied by defects of their projections to the striatum [1,2,3] and the presence of Lewy Bodies (LBs) in the remaining dopaminergic neurons [4,5]. LBs are insoluble intracellular inclusions formed mainly from misfolded α-synuclein (α-syn) oligomers [6], whereas normal α-syn located at the presynaptic terminals has the properties of regulating neurotransmitter release and synaptic plasticity [7,8]. The accumulation of LBs contributes to the destruction of the ubiquitin–proteasome system (UPS) [9,10,11] and the dysfunction of mitochondria and the lysosome, which causes dopaminergic neurons to be vulnerable to death and motor symptoms of PD [11,12]. Meanwhile, it is believed that pathological α-syn has the ability to spread to other non-dopaminergic nuclei, such as the raphe nucleus, hippocampus and amygdala, leading to non-motor symptoms in the progression of the disease [13,14,15]. The cardinal motor symptoms of PD include resting tremors, gait impairment and rigidity [16,17,18], while the non-motor manifestations cover sleep behavior disorder, urinary dysfunction, cognitive decline and depression [19,20]. However, effective treatments to alleviate these symptoms and disease progression are still lacking due to the side effects of therapeutic drugs and incomplete understanding about pathological mechanisms of PD [21,22,23].

HDAC6, one kind of class IIb HDACs, is highly expressed in the brain and germline tissue compared with other somatic tissues [24,25,26], and participates in a large number of physiological functions [27]. Meanwhile, the location of the *Hdac6* gene is on the X chromosome both in mice and human genomes [28,29]. Different from other HDACs, the structure of HDAC6 contains two independent and highly conserved catalytic domains for the deacetylation primarily of non-histone substrates in the cytoplasm as a result of regulation by the other three functional domains [30,31,32]. For example, the nuclear export signal (NES) domain helps guide HDAC6 to localize at the cytoplasm, while enzyme retention is associated with the Ser-Glu-containing tetrapeptide (SE14) domain and acetylated nuclear localization signal (NLS) domain [33,34]. In addition, the ubiquitin-binding zinc-finger (ZnF-UBP) domain makes HDAC6 more unique because of its function to recognize and help degrade ubiquitinated proteins via the autophagy pathway [24].

In recent years, many studies have revealed that HDAC6 is implicated in multiple neurodegenerative diseases [35,36,37] including PD. Inhibition of this enzyme is more likely to exert neuroprotection through promoting the survival and regeneration of neurons [38,39,40]. Neuroinflammatory reactions accompany the PD progression [41,42], as evidenced by inflammatory mediators and activated glial cells in the SN of postmortem PD patients and animal models [43,44]. There is some evidence suggesting that deacetylase activity of HDAC6 promotes inflammatory responses [45,46]; conversely, pharmacological inhibition of HDAC6 appears to attenuate inflammation in the progression of multiple neurodegenerative diseases or conditions such as PD, Alzheimer’s disease and Huntington’s disease [47,48,49,50], or retinal injury [51]. In addition, HDAC6 mediates the activation of the NLRP3 inflammasome, and the knockout or inhibition of HDAC6 suppresses inflammatory responses in vitro or in vivo [52,53] and attenuates 6-OHDA-induced dopaminergic neuronal loss in mice [53]. In addition to anti-inflammatory effects, inhibition of deacetylation activity of HDAC6 can also limit its modification to α-tubulin or microtubules, therefore increasing the acetylated level of tubulin and the axonal transport function and alleviating damage in PD models [54,55,56,57]. However, a few studies have reported the advantage of the deacetylation function of HDAC6 because it can modify heat shock proteins90 (Hsp90) or cortactin to promote the autophagy pathway [58,59,60]. On the other hand, more and more evidence showing the co-localization of HDAC6, ubiquitin and LBs supports the idea that HDAC6 assists in reducing cytotoxicity by degrading misfolded α-syn aggregates through the aggresome–autophagy pathway [58,61,62]. How the dual roles of anti-aggregation and pro-inflammation of HDAC6 are balanced in the progression of PD is worth exploring before it becomes a potential therapeutic target for PD [35,63,64].

In this study, the behaviors of mice with HDAC6 deficiency (KO) and control wild-type (WT) mice were analyzed, and the PD-related behaviors and nigrostriatal pathways of KO and WT mice of both genders were further investigated after an acute regimen of MPTP administration. The results showed that a lack of HDAC6 had moderate effects on behaviors and PD-like pathology induced by MPTP in male mice; however, HDAC6 deficiency led to a more pronounced protection in female PD mice.

## 2. Results

### 2.1. The Construction Strategy and Validation of Hdac6^−/−^ Mice

The construction strategy of *Hdac6*^−/−^ mice was to induce an early termination of translation by targeting the exon 11–14 of the mouse *Hdac6* gene using the CRISPR-Cas9 technique; the positions of primers for genotyping were also indicated (Figure S1a). Identification of *Hdac6*^−/−^ mice was performed at genome, transcription and protein levels (Figure S1b–d). The results of the qPCR and western blot showed that *Hdac6* mRNA and HDAC6 proteins were undetectable in the brain of *Hdac6*^−/−^ mice, respectively (Figure S1c,d). A major HDAC6 substrate is α-tubulin acetylated at lysine 40 (K40ac). HDAC6 deficiency increased striatal α-tubulin K40ac levels (Figure S1e), similar to the results by Govindajaran et al. [65]. Moreover, a loss of HDAC6 had no effect on the expression of other HDACs including *Hdac1* (Class I), *Hdac4* (Class IIa) and *Hdac11* (Class IV) (Figure S2). The above results proved that *Hdac6* knockout mice were successfully generated.

### 2.2. The Lack of HDAC6 Affects Animal Behaviors

Previous studies have shown that HDAC6 is involved in mood regulation [66,67] and the absence of functional HDAC6 has an anti-depressive effect in rodents [68,69,70]. Anxiety and depression are clinical non-motor symptoms of PD patients, which may affect the pathological progression of the disease [71,72]. In order to know the influence of HDAC6 depletion on animal mood, emotion-related behaviors of WT and KO mice of both genders were tested.

**Figure 1:**
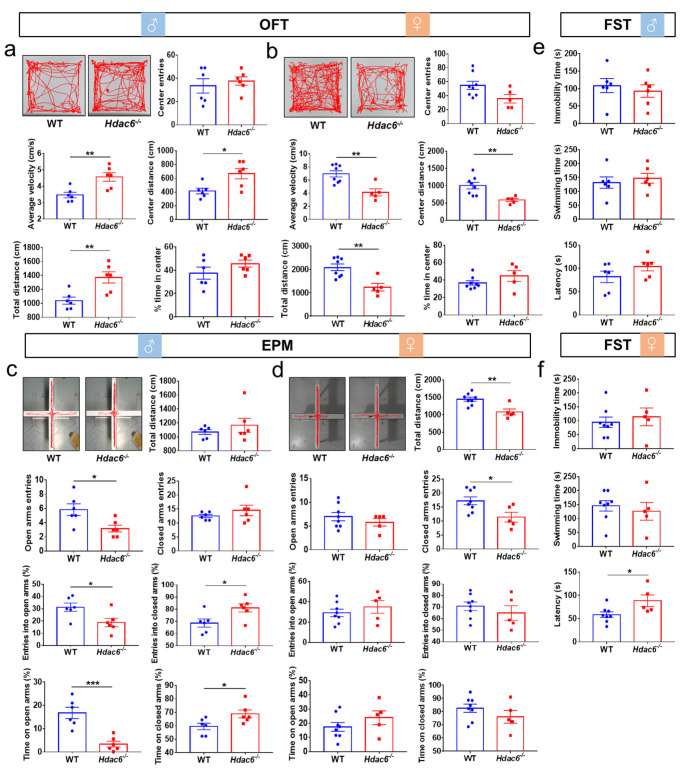
The effects of HDAC6 loss on the behaviors in mice. (**a**,**b**) The result of open field test (OFT) in male (**a**) and female (**b**) WT mice and *Hdac6*^−/−^ mice. (**c**,**d**) The result of elevated plus maze (EPM) in male (**c**) and female (**d**) WT mice and *Hdac6*^−/−^ mice. (**e**,**f**) The results of forced swimming test (FST) in male (**e**) and female (**f**) WT mice and *Hdac6*^−/−^ mice. *n* = 5–8, * *p* < 0.05, ** *p* < 0.01, *** *p* < 0.001.

In the open field test (OFT), compared with WT mice, male *Hdac6*^−/−^ mice showed a significant increase in the average speed of movement and the moving distance in the central region as well as in the whole area, whereas female mice exhibited the opposite performance, indicated by the dramatic decrease in the average speed, the total distance and the central distance. However, the time mice spent in the central area and the times they entered the center were unaffected in both male and female mice with HDAC deficiency (Figure 1a,b). The results from the elevated plus maze (EPM) test showed that the lack of HDAC6 significantly decreased the frequency of male mice entering the open arms, as well as the percentage of entries to the open arms to total entries and time spent inside the open arms. Conversely, there was an increase in the percentage of entries to the closed arms and the time spent inside them. In female mutant mice, we observed a decrease in the total distance traveled and the frequency of entries to the closed arms (Figure 1c,d). In the forced swimming test (FST), no effect of a HDAC6 loss on the latency, swimming time or immobility time was observed in male mice, but HDAC6 deficiency prolonged the latency in female mice (Figure 1e,f). Taken together, it was speculated that HDAC6 deficiency increased the activity of male mice and decreased that of female mice. Furthermore, male *Hdac6*^−/−^ mice showed a certain degree of anxiety in the EPM test, while female *Hdac6*^−/−^ mice exhibited a reduced level of depression to some extent in the FST.

### 2.3. The Lack of HDAC6 Does Not Affect the Contents of NE, DA and 5-HT in the Brain

**Figure 2:**
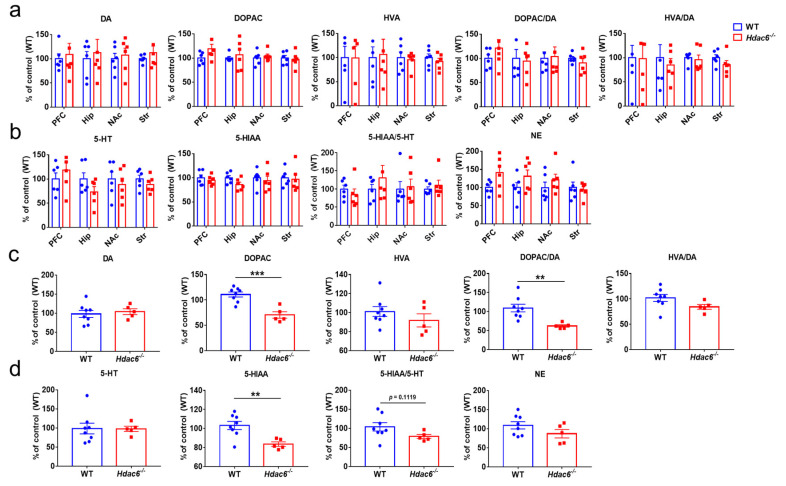
The effects of HDAC6 deficiency on the DA, 5-HT and NE contents in the brain of mice. (**a**,**b**) The levels of monoamine neurotransmitters in the prefrontal cortex (PFC), nucleus accumbens (NAc), hippocampus (Hip) and striatum (Str) of male WT and *Hdac6*^−/−^ mice were analyzed by HPLC; the ratios of DOPAC to DA, HVA to DA and 5-HIAA to 5-HT were also shown. (**c**,**d**) The levels of monoamine neurotransmitters in the striatum of female WT and *Hdac6*^−/−^ mice, and the ratios of DOPAC to DA, HVA to DA and 5-HIAA to 5-HT are shown. *n* = 5–8, ** *p* < 0.01, *** *p* < 0.001.

It has been observed that HDAC6 deficiency may cause anxiety-like behavior in males and a slight anti-depressive effect in females. As is known, the occurrence of anxiety and depression is often associated with the changes of monoamine neurotransmitters including dopamine (DA), serotonin (5-HT) and norepinephrine (NE) in the relevant brain regions such as the prefrontal cortex (PFC), nucleus accumbens (NAc), hippocampus (Hip) and striatum (Str) [73,74,75,76,77,78]. Therefore, monoamine levels in the above brain regions were detected in both KO and WT mice of different genders. To our surprise, HPLC results showed no change in the contents and metabolic levels of DA, 5-HT and NE in the PFC, NAc, Hip and striatum in male mice regardless of the presence of HDAC6 or not (Figure 2a,b). The results in female mice were different from those in male mice, mainly in terms of the striatal metabolism of DA and 5-HT. The contents of 3,4-dihydroxyphenylacetic acid (DOPAC), 5-hydroxyindoleacetic acid (5-HIAA) and the ratio of DOPAC to DA were significantly decreased in female mice, indicating the metabolic rate of DA decreased (Figure 2c,d).

### 2.4. HDAC6 Deficiency Slightly Alleviates the Motor Deficits Induced by MPTP

**Figure 3:**
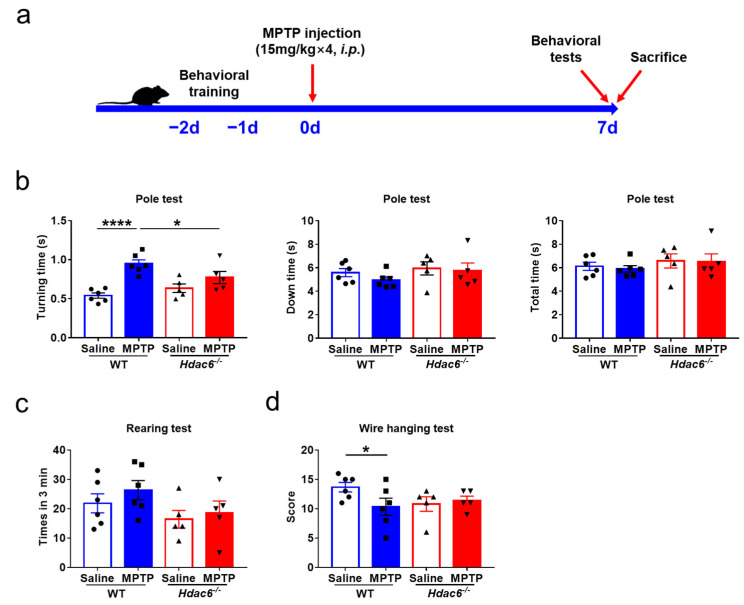
The effects of HDAC6 loss on PD-related motor injuries induced by MPTP injection in male WT and *Hdac6*^−/−^ mice. (**a**) The experimental schedule. (**b**–**d**) PD-related behavioral tests were performed on mice at 7 days after the last injection of MPTP or NS. (**b**) The results of pole test. (**c**) The results of rearing test. (**d**) The results of wire hanging test. *n* = 5–6, * *p* < 0.05, **** *p* < 0.0001.

Considering gender differences and the alterations of monoamine neurotransmitters caused by HDAC6 deficiency in female mice, both genders of mice were used for further exploration of the relationship between HDAC6 and PD. An acute PD model was constructed with intraperitoneal injection of MPTP, while the tests of PD-related behaviors were carried out at 7 days after the injection (Figure 3a and Figure S3a).

In the pole test, the turning time of MPTP-treated male and female WT mice increased significantly compared to their respective control WT mice and MPTP-treated KO mice (Figure 3b and Figure S3b); however, there were no differences in the climbing down and total climbing time between the two genotypes of male mice (Figure 3b), whereas total climbing time in MPTP-treated female KO mice was significantly reduced (Figure S3b). In the wire hanging test, the scores of male WT mice were significantly reduced after MPTP administration, indicating that the upper limbs’ strength was markedly decreased, whereas the absence of HDAC6 did not affect the performance of MPTP-challenged mice of both genders (Figure 3d and Figure S3d). Meanwhile, MPTP injection did not change the upper limbs’ coordination of WT and KO mice in the rearing test (Figure 3c and Figure S3c). Therefore, the results of behavioral tests indicated that HDAC6 depletion slightly alleviates PD-related motor deficits induced by MPTP.

### 2.5. HDAC6 Deficiency Does Not Affect MPTP-Induced Changes of Monoamine Neurotransmitters in the Striatum of Mice

**Figure 4:**
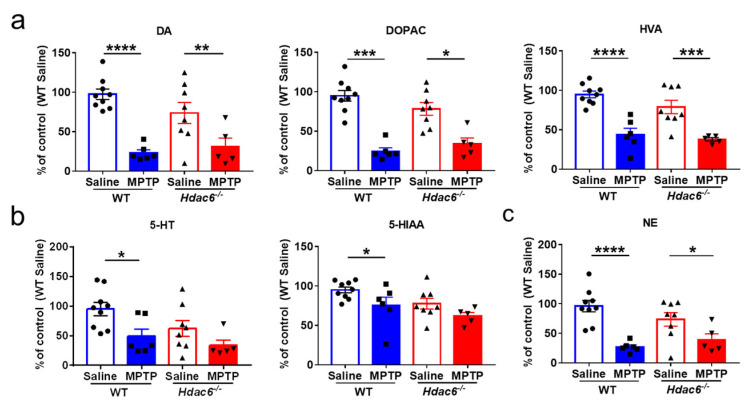
Effects of HDAC6 loss on the changes of monoamine neurotransmitters in the striatum of male WT and *Hdac6*^−/−^ mice at 7 days after MPTP injection. (**a**) The levels of DA and its metabolites DOPAC and HVA. (**b**) The levels of 5-HT and its metabolite 5-HIAA. (**c**) The levels of NE. *n* = 5–6, * *p* < 0.05, ** *p* < 0.01, *** *p* < 0.001, **** *p* < 0.0001.

To know whether the absence of HDAC6 had any effects on MPTP-induced changes of monoamine neurotransmitters, we conducted HPLC assays in the striatal tissues of mice. MPTP insult led to significant decreases in DA, DOPAC, HVA and NE in WT and KO mice, as well as 5-HT and 5-HIAA in WT mice. There were no differences between the two genotypes of mice after MPTP administration (Figure 4).

### 2.6. HDAC6 Deficiency Does Not Affect the MPTP-Induced Damages to the Nigrostriatal Pathway in Male Mice, but Attenuates the Depletion in Striatal TH Proteins in Female Mice

**Figure 5:**
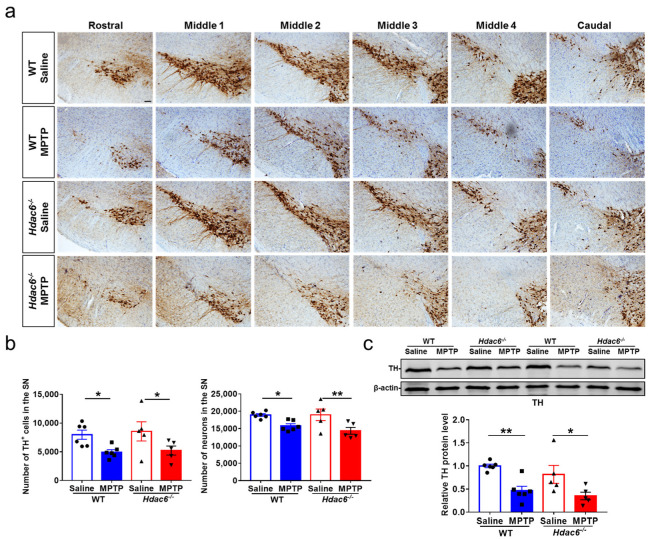
The impact of HDAC6 deficiency on MPTP-induced damages to the nigrostriatal dopaminergic system in male mice at 7 days after MPTP administration. (**a**,**b**) Immunohistochemical and Nissl staining (**a**) and counting of TH-positive and Nissl-positive cells (**b**) in the SNpc. Scale bar: 50 μm. *n* = 5–6. (**c**) Representative western blot of TH proteins in the striatum and quantification of TH protein levels; β-actin served as the control. *n* = 5–6, * *p* < 0.05, ** *p* < 0.01.

The homeostasis of monoamine neurotransmitters is maintained by the constant number of DA neurons and synaptic activity [79,80]. With immunohistochemical staining (IHC) and Nissl staining, as well as cell counting, we found that the reduction in the number of dopaminergic neurons and total neurons in the SNpc induced by MPTP intoxication was not influenced by HDAC6 deficiency in male mice (Figure 5a,b). In the striatum, the levels of TH proteins were significantly decreased in MPTP-treated WT and KO mice; there was no difference between the two genotypes of male mice (Figure 5c). However, in MPTP-treated female WT mice, the levels of striatal TH proteins were significantly reduced compared to the control WT mice and MPTP-treated female KO mice (Figure S4a,b). Therefore, the impact of HDAC6 deficiency on MPTP-induced damages to the nigrostriatal dopaminergic system has a certain difference in male and female mice.

### 2.7. HDAC6 Depletion Does Not Influence MPTP-Induced Glial Activation

**Figure 6:**
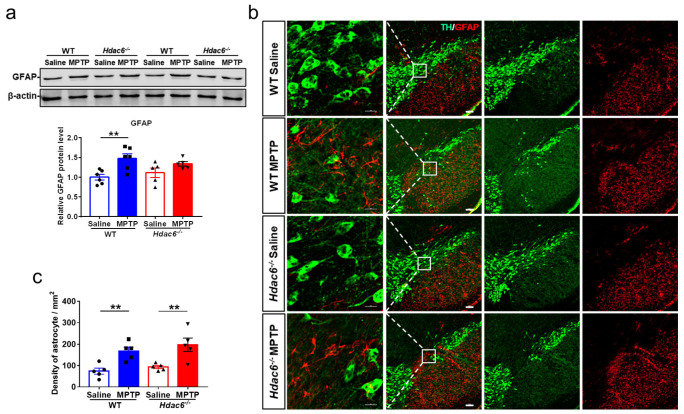
HDAC6 depletion impedes MPTP-induced astrocyte activation in the striatum, but not the SNpc of male mice at 7 days after MPTP administration. (**a**) Western blot showing levels of GFAP proteins in the striatum. β-actin served as the control. *n* = 5–6. (**b**) Representative double immunofluorescence staining for TH (green) and GFAP (red) in the SN. Scale bar: 100 μm. (**c**) Quantification of GFAP-positive cells in the SNpc. *n* = 5–6, ** *p* < 0.01.

**Figure 7:**
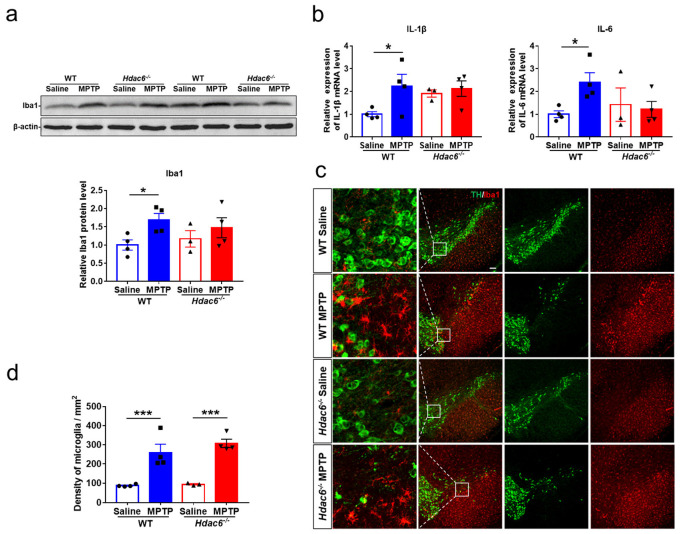
HDAC6 depletion impedes MPTP-induced microglia activation in the striatum, but not the SNpc of male mice at 3 days after MPTP administration. (**a**) Western blot showing levels of Iba1 proteins in the striatum. β-actin served as the control. (**b**) The transcriptional levels of *IL-1β* and *IL-6* in the striatum. (**c**) Double immunofluorescence staining for TH (green) and Iba1 (red) in the SN. Scale bar: 100 μm. (**d**) Counting of Iba1-positive cells in the SNpc. *n* = 3–4. * *p* < 0.05, *** *p* < 0.001.

Neuroinflammation may exacerbate the progression of PD [41,81,82]. MPTP administration elicited an upregulation in GFAP proteins in the striatum of male WT, but not male KO mice (Figure 6a). By the immunofluorescence staining and cell counting, we found the number of GFAP-positive cells in the SNpc of both WT and KO mice was significantly increased after MPTP injection (Figure 6b,c). The activation of microglia usually precedes the activation of astrocytes [83]. Microglia activation was analyzed at 3 days post injection. MPTP administration upregulated the protein levels of Iba1 and the transcriptional levels of *IL-1β* and *IL-6* in the striatum of male WT, but not male KO mice (Figure 7a,b). The number of Iba1-positive cells in the SNpc of both WT and KO mice treated with MPTP was significantly increased (Figure 7c). Notably, there was no difference in the striatal levels of GFAP and Iba1 proteins among all the four female groups at 7 days post injection (Figure S4a,c,d).

### 2.8. HDAC6 Deficiency Does Not Affect Inflammatory or Apoptotic Pathways in the Striatum of MPTP-Induced PD Mice

**Figure 8:**
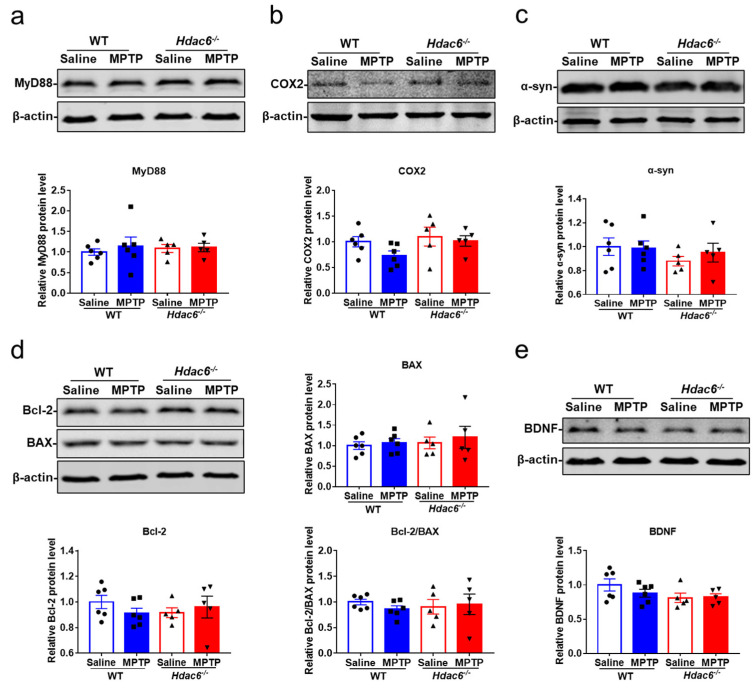
The effects of HDAC6 depletion on the expression of inflammatory, apoptotic and neurotrophic molecules in the striatum of male mice at 7 days after MPTP administration. (**a**–**e**) Representative western blot showing the protein levels of MyD88 (**a**), COX2 (**b**), α-syn (**c**), Bax and Bcl-2 (**d**) and BDNF (**e**) in the striatum. The ratio of Bcl-2 to Bax is also shown. *n* = 5–6.

The expression of molecules associated with inflammatory pathways in the striatum was further investigated. Our results showed that the levels of COX2 and MyD88 proteins in PD mice were not influenced by HDAC6 deficiency (Figure 8a,b). Owing to the fact that HDAC6 is involved in the autophagy pathway to remove abnormal accumulation of proteins in cells, such as α-syn in PD, the expression of α-syn proteins in the striatum was detected, but there were no differences among all groups (Figure 8c). Moreover, we explored the apoptotic signaling pathway, since autophagy and apoptosis, as two coordinated modes of cell death, both occur in PD [84,85]. HDAC6 depletion had no effect on the expression of apoptosis-related proteins, such as B cell leukemia/lymphoma-2 (Bcl-2) and Bcl-2-associated X (Bax), in the striatum of PD mice (Figure 8d). The striatal levels of brain-derived trophic factor (BDNF) also did not differ in the four experimental groups of mice (Figure 8e). These results suggest that the expression of molecules related to inflammatory or apoptotic pathways in the striatum was not affected by HDAC6 deficiency.

## 3. Discussion

In this study, the behaviors and Parkinson’s disease-like pathology in HDAC6 deficient mice were analyzed (Table S1). Based on the results of all the behavioral tests, we found that the effects of a HDAC6 loss on animal neuropsychiatric behaviors are gender-specific: male mice are hyperactive and anxious to some extent, while female mice are appreciably hypoactive and anti-depressive. The hyperactivity of male mice is also reported in other studies [69,86]. However, the phenomenon of anxiety is inconsistent; some reports demonstrate that knockout of *Hdac6* might have an anxiolytic-like effect on mice [68,69], while another supports our results, in which an increase in anxiety-like behavior is reported for grouped male and female *Hdac6* null mice [87]. FST is commonly used to detect depression in animals; our findings suggest that the effect of HDAC6 deficiency on depression in animals was subtle, which is in agreement with the result of a tail suspension test (TST) in neuron-specific *Hdac6* knockout mice [88]. In addition, HDAC6 deficiency, particularly in serotoninergic neurons, might produce modest anti-depressive effects in TST [68]. Grouped male and female young mice (4 weeks old) also exhibited as less depressive in the absence of HDAC6 [87]. Therefore, the effect of HDAC6 deficiency on neuropsychiatric behaviors in animals is complex.

In addition to animal behaviors, sex differences were observed in the metabolism of monoamine neurotransmitters. In female mice, the contents of DA and 5-HT were not affected by a HDAC6 loss, but the levels of their metabolites and the metabolic rate of DA were significantly decreased, which might contribute to the mild anti-depressive mood observed in this study. Fukada et al. reported that there were no differences in the metabolism of monoamine neurotransmitters in the striatum of male *Hdac6*^−/−^ mice [86], which coincides with our findings. In addition to the striatum, we also found there were no changes in the metabolism of monoamine neurotransmitters in other brain regions associated with emotion in male KO mice, which is close to the study by Espallergues et al. [68].

Both genders of mice were further used as experimental animals to establish an acute MPTP-induced PD model. Afterwards, the relationship between HDAC6 depletion and the pathological progress of PD was investigated. In the evaluation of behaviors related to PD, both male and female WT mice treated with MPTP exhibited a prolonged turning time in the pole test, only male WT mice showed a lower score in the wire hanging test after MPTP administration and there were no dramatic changes in the rearing test, whereas both male and female KO mice exhibited no significant difference in any of the three behavioral tests after MPTP administration. Moreover, the striatal TH protein level in female KO mice was obviously higher than that of the WT counterpart after MPTP injection. These results suggest that the depletion of HDAC6 leads to protection against MPTP-induced motor injury in mice. Suppression of HDAC6 alleviates motor injury of PD mice in the rotarod test and the pole test, and such an alleviating phenomenon also appears in PD models of drosophila and zebrafish [49,54,89]. On the other hand, a few studies report that deficiency or inhibition of HDAC6 aggravates dyskinesia in PD animals induced by UPS toxins [58,90]. In this study, the climbing down in the pole test did not change among all groups, similar to our previous studies [83,91]. Therefore, it is speculated that the MPTP-induced PD model and the protein aggregation-induced PD vary in their sensitivity to different behavioral paradigms.

In the analysis of the glial activation and the expression of inflammatory molecules in the nigrostriatal pathway, we found that except for the different changes in the striatal GFAP and Iba1 proteins, and the *IL-1β* and *IL-6* transcripts between male WT and KO mice treated with MPTP, the numbers of GFAP^+^ astrocytes and Iba1^+^ microglia in the SNpc and the striatal levels of COX2 and MyD88 did not differ between the two genotypes of mice. Despite a growing body of evidence demonstrating that HDAC6 inhibition could play an anti-inflammatory role in PD [47], our results suggest that the effect of HDAC6 in neuroinflammation might be limited in a MPTP-induced mouse PD model.

Since HDAC6 mediates the clearance of the abnormal accumulated proteins within cells by autophagy, we detected the striatal α-syn proteins. The expression of α-syn proteins is not upregulated in mice injured by an acute regimen of MPTP, and the levels of α-syn proteins do not alter in KO mice as well. Interestingly, in PD models with α-syn overexpression, the deficiency or inhibition of HDAC6 actually accelerates PD progression [58,90,92].

Autophagy and apoptosis can inhibit each other, and there are opinions suggesting the inhibition of HDAC6 leads to apoptosis by inhibiting autophagy [93,94,95,96]. On the other hand, growing evidence exists that HDAC6 inhibition can suppress intracellular inflammatory responses associated with mitochondrial dysfunction or microglial activation, thus exerting an anti-apoptotic effect [49,97,98,99,100]. In this study, we did not detect any changes in the expression of the apoptosis-related proteins Bcl-2 and Bax, and pro-survival neurotrophic factor BDNF, in the striatum of both WT and KO mice after MPTP injection. One possible explanation is the PD models used are different.

In conclusion, the absence of HDAC6 leads to a gender-specific alteration in behaviors, and has moderate effects on the pathology of PD mice.

## 4. Materials and Methods

### 4.1. Animals

Male and female *Hdac6*^−/−^ mice (generated by the CRISPR-Cas9 gene editing technology) and wild-type (WT) mice in the background of C57BL/6J, 10–15 weeks of age and 24–28 g, or female *Hdac6*^−/−^ mice and WT mice, 21–25 weeks of age, were obtained from the Shanghai Model Organisms Center (Shanghai, China). Animals were group-housed with free access to water and food in a 12–12 h light/dark cycle with room temperature at 22 ± 2 °C and humidity of 55 ± 5%.

### 4.2. MPTP Treatments of Mice

Mice were injected intraperitoneally (*i.p.*) with MPTP (1-methyl-4-phenyl-1,2,3,6-tetrahydropyridine) (Sigma, Saint Louis, MO, USA) or normal saline (NS) in an acute regimen of 15 mg/kg for 4 doses at 2 h intervals. Behavioral experiments related to PD were carried out at 7 days after MPTP administration for the evaluation of general motor function and then mice were sacrificed after perfusion of 0.9% NS. PFC, NAc, Hip, striatum and midbrain were carefully dissected for subsequent experiments.

### 4.3. Pole Test

Briefly, mouse was placed with head up on the top of a rough-surface wooden pole (1.5 cm in diameter and 50 cm in height), which stood vertically in a clean cage. When the mouse started to turn its head around, it was recorded as the beginning of turning around (T1). When the mouse completely turned its body downward and was ready to climb down, it was recorded as the end of turning (T2), namely the beginning of down time. When the mouse touched the bottom of the pole, it was recorded as the end of descending (T3). The total time was the sum of turning time (T2-T1) and down time (T3-T2). Each mouse was trained for 2 days before MPTP treatment, and in the formal test, the experiment was repeated three times to obtain an average of the recorded time.

### 4.4. Rearing Test

This test was used to assess upper limbs’ coordination and spontaneous mobility. Mice were placed in a transparent beaker (10 cm in diameter and 20 cm in height) and the number of times they stood up on their hind legs with forelimbs against the wall over a 3-minute period was counted.

### 4.5. Wire Hanging Test

Mice were placed with their forelimbs on a horizontally suspended wire (2 mm in diameter and 50 cm in length) and the number of times they climbed from the center of the wire to the platforms (40 cm in height) on either side within 3 min was recorded, as well as the number of times they fell. The base score was 10 points; one point was added for climbing on the platform, and one point was deducted for falling off the wire.

### 4.6. Open Field Test (OFT)

Each mouse was placed individually at the center of the OF apparatus (40 × 40 × 40 cm) and allowed to move freely for 5 min. During this time, the total distance, average speed of movement, frequency and time of entry into the central area were all recorded by the tracking camera at the top of the arena and analyzed by the Noldus EthoVision XT software.

### 4.7. Elevated Plus Maze Test (EPM)

The implementation of EPM test was used to assess anxiety in animals. Animals were placed in the EPM apparatus near the intersection (10 × 10 cm) of the open and enclosed arms (50 × 10 × 0.5 cm), and allowed to move freely in the device for 5 min. The total distance and time of the movement in the open and enclosed arms, as well as the number of entries into each arm, were measured and analyzed by the Noldus EthoVision XT software.

### 4.8. Forced Swimming Test (FST)

Depressive state in rodents was assessed using FST. In this experiment, mice were placed head down in a transparent cylinder (15 cm in diameter and 24.5 cm in height) filled with water (25 ± 1 °C) to a depth of about 15 cm. Mice were forced to swim for 6 min and the latency to the first immobile state and the cumulative immobile time within the last 4 min were recorded. Stopping to struggle in the water and making only slight movements to keep the head afloat for breathing was considered immobile state.

### 4.9. High-Performance Liquid Chromatography (HPLC)

The tissues, such as PFC, NAc, Hip and striatum, were homogenized with 0.4 M cold perchloric acid (HClO_4_) at the ratio of 1 mg tissue to 10 μL liquid. Then, the supernatant solution was collected with centrifugation at 12,000 rpm for 15 min at 4 °C, and the contents of DA, 5-HT, 3,4-dihydroxyphenylacetic acid (DOPAC), homovanillic acid (HVA) and 5-hydroxyindoleacetic acid (5-HIAA) in the samples were detected by chromatograph (ESA, Chelmsford, MA, USA).

### 4.10. RNA Extraction and Quantitative Real-Time PCR

Total RNA was extracted from brain using TRIzol reagent (Tiangen Biotech, Beijing, China) and the concentrations were measured using a multifunctional MicroplateReader (BioTek instruments, Winooski, VT, USA). A total of 1 µg RNA was converted into cDNA using a high-capacity cDNA Reverse Transcription Kit (Tiangen Biotech, Beijing, China) according to the manufacturer’s instructions. The resulting cDNA was used to detect the relative mRNA levels by real-time PCR using a quantitative thermal cycler (Mastercycler ep realplex, Eppendorf Innovation Company, Hamburg, Germany). Following PCR conditions were used: 95 °C for 3 min, followed by 40 cycles at 95 °C for 15 s, 58 °C for 15 s and 72 °C for 20 s, as well as a final extension at 60 °C for 1 min. Relative gene expression values were calculated with the 2^−ΔΔCt^ algorithm and samples were normalized to β-actin genes. The primers used are shown as following: *Hdac6*: 5′-ATGGTCGCTTCTGGCCCC-3′ and 5′-CAGCCATCTCTCCCTTGGG-3′; *β-actin*: 5′-CAGGATGCAGAAGGAGATTAC-3′ and 5′-AACGCAGCTCAGTAACAGTC-3′.

### 4.11. Protein Extraction and Western Blot

The striatum tissues were lysed with 200 μL cold RIPA lysis buffer, which contains phosphatase inhibitors and protease inhibitors, and the concentration of the extracted proteins was measured by the BCA assay (Beyotime Biotechnology, Shanghai, China). After mixing with loading buffer and boiling, the proteins (30 μg) were separated by 10–12.5% sodium dodecyl sulfate–polyacrylamide gel electrophoresis (SDS-PAGE) gels and transferred to polyvinylidene fluoride (PVDF) membranes. The membranes were blocked with 5% skim milk for 1 h and then incubated at room temperature with primary antibody for 1 h and at 4 °C overnight. After washing, the blots were immersed in the fluorescent secondary antibody (LI-COR, Lincoln, NE, USA) for 1 h at room temperature. Then, WB images were captured with an Odyssey infrared imaging system (LI-COR, USA), and ImageJ 1.x (National Institutes of Health, Bethesda, MD, USA) software was used to quantify densitometry of the protein bands. The primary antibodies used were as follows: mouse anti-TH antibody (1:1000; Abcam, Boston, MA, USA), rabbit anti-GFAP antibody (1:1000; Proteintech, Wuhan, China), rabbit anti-Iba1 antibody (1:1000; Abcam, USA), mouse anti-β-actin antibody (1:2000; Santa Cruz Biotechnology, Santa Cruz, CA, USA), rabbit anti-MyD88 antibody (1:1000; Affinity Biosciences, Liyang, China), rabbit anti-BDNF antibody (1:1000; Santa Cruz, USA), rabbit anti-COX2 antibody (1:1000; Abcam, USA), mouse anti-α-syn (1:1000; Abcam, USA), rabbit anti-Bax (1:1000; Cell Signaling Technology, Danvers, MA, USA), rabbit anti-Bcl2 (1:1000; Cell signaling technology, USA), rabbit anti-HDAC6 (1:1000; Affinity, USA) and rabbit anti-Acetyl-alpha Tubulin (1:1000; Affinity, USA).

### 4.12. Immunofluorescence and Immunohistochemistry Staining

Mice midbrains were fixed with 4% paraformaldehyde for 24 h and dehydrated with 20% and 30% sucrose, respectively, for 24 h at 4 °C. After that, midbrains were embedded in optimal cutting temperature compound (Sakura, Torrance, CA, USA) and serially cut into 30 μm frozen coronal sections by freezing cryostat (Leica, Wetzlar, Germany). For immunofluorescence staining, brain sections were blocked with phosphate-buffered saline (PBS) containing 10% normal goat serum and 0.2% Triton X-100 at 37 °C for 1 h and incubated with primary mouse anti-TH antibody (1:1000; Abcam, USA), rabbit anti-GFAP antibody (1:1000; Proteintech, China) and rabbit anti-Iba1 antibody (1:1000; Abcam, USA) at 4 °C overnight. After washing with PBST (containing 0.05% Triton in PBS), the sections were incubated with appropriate secondary antibodies: Alexa Fluor 488 and 594 (1:1000; Invitrogen, Waltham, MA, USA) for 1 h at 37 °C. Images were captured with a Leica confocal microscope TCS SP-2 (Leica, Wetzlar, Germany). For immunohistochemistry staining, slides were treated with 0.3% H_2_O_2_ to quench endogenous peroxidases and 0.01 M sodium citrate solution (pH = 6.0) for antigen retrieval and then blocked with PBS (containing 10% normal goat serum and 0.2% Triton X-100) for 45 min at 37 °C. The slides were incubated with primary mouse anti-TH antibody (1:1000; Abcam, USA) at 4 °C overnight and then with biotinylated anti-mouse secondary antibodies (1:200; Vector Laboratories, Burlingame, CA, USA) and AB peroxidase (1:200; Vector Laboratories, USA) in turn for 45 min at 37 °C after washing. DAB kit (Vector Laboratories, USA) was used to detect the peroxidase and images were obtained with a microscope (Olympus, Tokyo, Japan). The brain slices were stained with 0.1% cresyl violet (Millipore, Billerica, MA, USA) for Nissl staining. For cell counting, Iba1-positive and GFAP-positive cells in the SNpc were counted using Image J 1.x software. Briefly, a total of 4 coronal sections were collected and counted in the regions of SNpc defined by TH staining according to published method [83]. TH^+^ cell counting and Nissl^+^ total neuron counting in the SNpc were conducted using a Stereo Investigator system (Micro Brightfield, Williston, VT, USA). A total of six sections (one out of every five sections) per mouse from Bregma −2.80 to −3.80 mm were collected and actually counted under a 20× objective.

### 4.13. Statistical Analysis

All data are presented as the mean ± SEM and were analyzed with Prism 7 (GraphPad Software Inc., San Diego, CA, USA). Statistical analysis among three or more groups was carried out using one-way ANOVA followed by Uncorrected Fisher’s LSD test or Uncorrected Dunn’s test and differences between two groups were analyzed by unpaired t-test or Mann–Whitney test. Two-way ANOVA with Uncorrected Fisher’s LSD test was used to compare statistical differences in the content of monoamine neurotransmitters in multiple brain regions. *p* < 0.05 was set to be statistically significant.

## Data Availability

The datasets used and analyzed during the current study are available from the corresponding authors upon request.

## Supplementary Materials

The following supporting information can be downloaded at: https://www.mdpi.com/article/10.3390/ijms24129975/s1.

## Abbreviations

The following abbreviations are used in this manuscript:

HDAC6	Histone deacetylase 6
PD	Parkinson’s disease
MPTP	1-methyl-4-phenyl-1,2,3,6-tetrahydropyridine
SNpc	Substantia nigra pars compacta
PFC	Prefrontal cortex
NAc	Nucleus accumbens
Hip	Hippocampus
Str	Striatum
LBs	Lewy Bodies
α-syn	α-synuclein
UPS	Ubiquitin–proteasome system
NES	Nuclear export signal
SE14	Ser-Glu-containing tetrapeptide
NLS	Nuclear localization signal
ZnF-UBP	Ubiquitin-binding zinc-finger
Hsp90	Heat shock proteins90
KO	HDAC6 deficiency
WT	Wild-type
OFT	Open field test
EPM	Elevated plus maze
FST	Forced swimming test
TST	Tail suspension test
5-HT	Serotonin
5-HIAA	5-hydroxyindoleacetic acid
DA	Dopamine
DOPAC	3,4-dihydroxyphenylacetic acid
NE	Norepinephrine
IHC	Immunohistochemical staining
IF	Immunofluorescence staining
SDS-PAGE	Sodium dodecyl sulfate–polyacrylamide gel electrophoresis
PVDF	Polyvinylidene fluoride
Bcl-2	B cell leukemia/lymphoma-2
Bax	Bcl-2-associated X
BDNF	Brain-derived trophic factor
