# Supplementary material for: HDAC6 Deficiency Has Moderate Effects on Behaviors and Parkinson’s Disease Pathology in Mice
Source: Int J Mol Sci. 2023 Jun 9;24(12):9975. doi: 10.3390/ijms24129975 (PMC10298689; doi:10.3390/ijms24129975)
Supplement: Supplementary file 1 [file ijms-24-09975-s001.zip › ijms-2328637-supplementary.pdf]

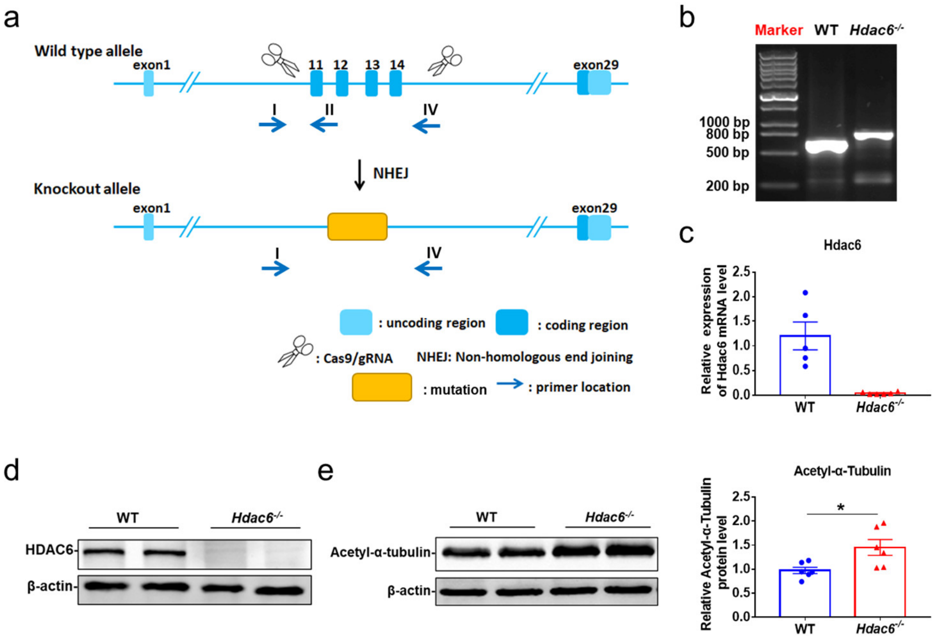

Figure S1. The targeting strategy and validation of HDAC6 deficiency in mice. (**a**) The construction strategy of *Hdac6*$^{-/-}$ mice. (**b**) Genotyping of *Hdac6*$^{-/-}$ mice. PCR primers used are indicated as primers I, II, IV. (**c**) Verification of HDAC6 deficiency in the brain of *Hdac6*$^{-/-}$ mice by qPCR. n=5-6. (**d**) Verification of HDAC6 deficiency in the brain of *Hdac6*$^{-/-}$ mice by Western blot. (**e**) The effect of HDAC6 deficiency on the acetylation of α-tubulin in the striatum of mice. n=6, *$p < 0.05$.

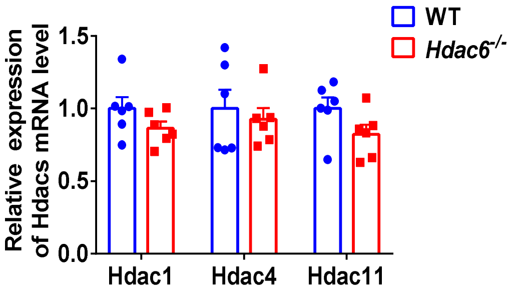

Figure S2. The transcripts of *Hdac1*, *Hdac4* and *Hdac11* in the brain of WT and *Hdac6*$^{-/-}$ mice detected by qPCR. n=6.

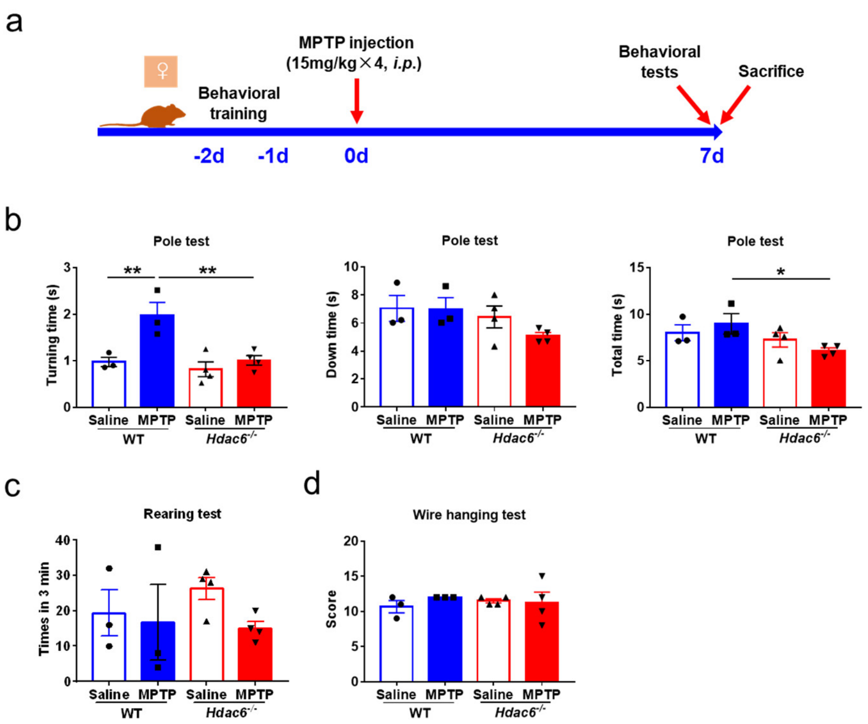

Figure S3. The effects of HDAC6 loss on PD-related motor injuries induced by MPTP injection in female WT and *Hdac6*$^{-/-}$ mice. (**a**) The experimental schedule. (**b, c, d**) PD-related behavioral tests were performed in mice at 7 days after the last injection of MPTP or NS. (**b**) The results of pole test. (**c**) The results of rearing test. (**d**) The results of wire hanging test. n=3-4, *$p < 0.05$, **$p < 0.01$.

.

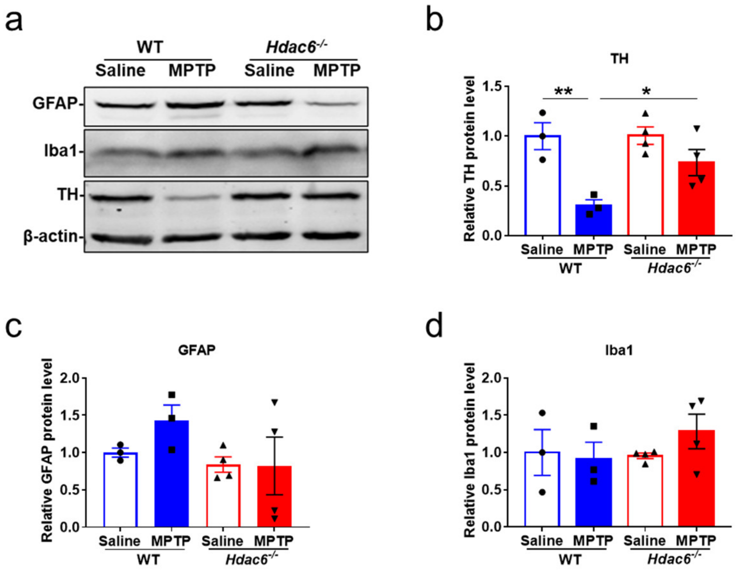

Figure S4. The impact of HDAC6 deficiency on MPTP-induced changes in the striatum of female WT and *Hdac6*$^{-/-}$ mice at 7 days after MPTP administration. (**a**) Representative western blot of striatal TH, GFAP and Iba1 proteins, β-actin served as the control. (**b-d**) Quantification of TH protein levels (**b**), GFAP protein levels (**c**), and Iba1 protein levels (**d**) . n=3-4, *$p < 0.05$, **$p < 0.01$.

Supplementary Table

Table S1. The main results

Direction	Index	Male				Female			
		WT		*Hdac6*$^{-/-}$		WT		*Hdac6*$^{-/-}$	
		Saline	MPTP	Saline	MPTP	Saline	MPTP	Saline	MPTP
Behaviors	Motor activity		○	↑	○		○	↓	○
	Anxiety or depression		○	↑	○		○	↓	○
PD-related behaviors	Motor ability		↓	-	-↑		↓	-	-↑
	Limb strength		↓	-	-		-	-	-
DA system	Striatal TH proteins		↓	-	↓		↓	-	-↑
Inflammation	Activation of astrocyte in SNpc (7d)		↑	-	↑		○	○	○
	Activation of microglia in SNpc (3d)		↑	-	↑		○	○	○
	Activation of astrocyte in striatum (7d)		↑	-	-		-	-	-
	Activation of microglia in striatum (3d)		↑	-	-		- (7d)	- (7d)	- (7d)

↑ ↓: enhancement or reduction vs. respective saline control; ↑ ↓: enhancement or reduction vs. WT saline control;

↑ : enhancement vs. WT MPTP group; - : no change vs. respective saline control; - : no change vs. WT saline control;

○: not tested.
